# Weed Species from Tea Gardens as a Source of Novel Aluminum Hyperaccumulators

**DOI:** 10.3390/plants12112129

**Published:** 2023-05-27

**Authors:** Roghieh Hajiboland, Aiuob Moradi, Ehsan Kahneh, Charlotte Poschenrieder, Fatemeh Nazari, Jelena Pavlovic, Roser Tolra, Seyed-Yahya Salehi-Lisar, Miroslav Nikolic

**Affiliations:** 1 Department of Plant, Cell and Molecular Biology, University of Tabriz, Tabriz 51666-16471, Iran; fatimanazari8@gmail.com (F.N.); y_salehi@tabrizu.ac.ir (S.-Y.S.-L.); 2 Watershed Management and Forests and Rangelands Research Department, Guilan Agricultural and Natural Resources Research and Education Center, Agricultural Research, Education and Extension Organization (AREEO), Rasht 41635-3394, Iran; aiuobmoradi50@gmail.com; 3 Tea Research Center, Iran Horticultural Science Research Institute, Agricultural Research, Education and Extension Organization (AREEO), Lahijan 44159-77788, Iran; kahneh_ehsan@yahoo.com; 4 Plant Physiology Laboratory, Bioscience Faculty, Universidad Autónoma de Barcelona, 08193 Bellaterra, Spain; charlotte.poschenrieder@uab.cat (C.P.); roser.tolra@uab.cat (R.T.); 5 Institute for Multidisciplinary Research, University of Belgrade, Kneza Viseslava 1, 11030 Belgrade, Serbia; jelena.pavlovic@imsi.bg.ac.rs

**Keywords:** acid soils, aluminum accumulation, heavy metals, iron, manganese, Northern Iran

## Abstract

Increased availability of toxic Al^3+^ is the main constraint limiting plant growth on acid soils. Plants adapted to acid soils, however, tolerate toxic Al^3+^, and some can accumulate Al in their aerial parts to a significant degree. Studies on Al-tolerant and Al-accumulating species have mainly focused on the vegetation of acid soils distributed as two global belts in the northern and southern hemispheres, while acid soils formed outside these regions have been largely neglected. The acid soils (pH 3.4–4.2) of the tea plantations in the south Caspian region of Northern Iran were surveyed over three seasons at two main locations. Aluminum and other mineral elements (including nutrients) were measured in 499 plant specimens representing 86 species from 43 families. Al accumulation exceeding the criterion for accumulator species (>1000 µg g^−1^ DW) was found in 36 species belonging to 23 families of herbaceous annual or perennial angiosperms, in addition to three bryophyte species. Besides Al, Fe accumulation (1026–5155 µg g^−1^ DW) was also observed in the accumulator species that exceeded the critical toxicity concentration, whereas no such accumulation was observed for Mn. The majority of analyzed accumulator plants (64%) were cosmopolitan or pluriregional species, with a considerable rate of Euro-Siberian elements (37%). Our findings, which may contribute to phylogenetic studies of Al accumulators, also suggest suitable accumulator and excluder species for the rehabilitation of acid-eroded soils and introduce new model species for investigating Al accumulation and exclusion mechanisms.

## 1. Introduction

Acid soils occupy approximately 30% (3950 Mha) of the world’s ice-free land area. Of this, about 67% is covered by forests and woodlands, 18% by savanna, prairie and steppe vegetation, and 4.5% (179 Mha) is used for crop cultivation. About 33 Mha of acid soils support the growth of perennial tropical crops such as tea [1]. The acid soils of the Earth occur in two global belts: the boreal cool temperature belt and the tropical belt [1]. Acid soil develops naturally when basic cations (Ca^2+^, Mg^2+^, K^+^, and Na^+^) are leached down the soil profile, lowering the pH of the soil solution. Another source of soil acidification is acid rain with sulfur dioxide and oxides of nitrogen caused by anthropogenic air pollution. Additionally, the use of ammonia and amide-containing fertilizers in farming practices can directly accelerate soil acidification [2]. It is also well established that the cultivation of N_2_ fixing leguminous crops acidifies soils because of their greater uptake of cations than anions and the consequent release of H^+^ into the soil [3]. Acid soils are characterized by high Al^3+^ activity, which induces a rhizotoxic effect on most plant species [4].

Aluminum is the most abundant mineral soil component and the third most abundant element in the Earth’s crust. In soil, it occurs in less toxic forms, such as aluminosilicates or aluminum oxides of the clay fraction. However, upon soil acidification, Al is released from the soil minerals and occurs primarily as [Al(H_2_O)_6_]^3+^, also expressed as Al^3+^. Globally, high Al^3+^ availability severely restricts crop production on acid soils [5].

Unlike the majority of crop species, which are either not or only poorly adapted to acid soil conditions, natural vegetation that has evolved under these conditions is perfectly adapted for survival and growth in the presence of toxic Al^3+^ concentrations [6]. These species employ two strategies to cope with high levels of active Al^3+^ in soil. The ‘avoidance or exclusion’ strategy operates by apoplastic detoxification mechanisms such as external chelation of Al^3+^ by the release of organic acids and phenolics from the roots, thereby preventing Al^3+^ uptake and entry into the root symplast. By contrast, plants able to manage high Al^3+^ concentrations accumulated in the symplast utilize the ‘tolerance strategy’, which involves internal sequestration and detoxification of Al^3+^ [7,8].

A high tolerance to tissue Al is found in the Al-accumulating species (or Al-hyperaccumulating species) with more than 1000 ppm Al (1000 µg g^−1^ dry weight) in dried leaf tissue [9]. According to this criterion, Al accumulation is found in approximately 45 plant families, including Proteaceae, Anisophylleaceae, Polygalaceae, Cunoniaceae and Rubiaceae, and several representatives of Laurales, Malpighiales, Myrtales, Ericales, and Aquifoliales. The vast majority are tropical or subtropical woody plants [9,10]. There are, however, several herbaceous Al accumulators, such as certain ferns and lycophytes [11], as well as the Angiosperms *Fagopyrum esculentum* [12] and *Plantago almogravensis* [13].

The ability to accumulate high levels of Al has arisen multiple times in flowering plants, and it has been lost independently in some more-evolved branches [9]. Al accumulation varies at diverse taxonomic levels; thus, analyses of a broader range of taxa are required to investigate the origin and taxonomic significance of Al accumulation in several clades [9]. Plant scientists will likely discover many more unidentified Al accumulators growing on acid and acidified soils [9].

Due to the increasing acidification of the world’s soils by acid rain and intensive farming, there has been a surge in interest in Al toxicity and accumulation in plants in recent years [14]. In addition, the destruction of forests and woodlands resulted in the loss of >250 million ha of forests, leaving vast anthropogenic savannas on heavily eroded acid soils [15,16]. For the revegetation and protection of deforested and abandoned areas, as well as the development of agriculture and agroforestry on degraded acid soils, a comprehensive understanding of plant species and families with high adaptability for growing on acid soils is required. In addition, identifying additional Al-tolerant and Al-accumulating species could aid in determining unexplored adaptation mechanisms and introduce new model species for internal detoxification and Al hyperaccumulation. For the latter purpose, it is necessary to consider the weed communities on acid soils in other biomes. In particular, little is known of the status of Al accumulation in weeds on acid soils formed outside the two global belts.

Soils in tea plantations are typically low in pH levels [17]. In Iran, tea gardens are primarily situated on the northern foothills of the Alborz Mountains. Although the soil pH in the natural forests of this region (Hyrcanian forests) is below 6.0 [18], the soil pH in tea gardens is further reduced to about 4.0 due to the long-term use of ammonium fertilizers and the inherent capacity of tea roots to acidify the soil. These low pH conditions may act as selective pressure and encourage the colonization of tea plantations by weed species tolerant to high Al^3+^ activity. According to our observations, a particular flora has taken root in abandoned and unmanaged gardens, and its investigation offers a rare opportunity to discover unstudied Al-accumulating species and new species with extreme Al tolerance. This study may contribute to introducing new Al accumulators to increase our understanding of Al accumulation in plant families where this trait has not yet been tested.

## 2. Results

A total of 86 species from 43 families were collected from two primary locations and their surrounding locations during six visits throughout the seasons of autumn, winter and spring (Table S1).

### 2.1. Al Concentration in the Specimens Collected from Acid Soils

**Figure 1:**
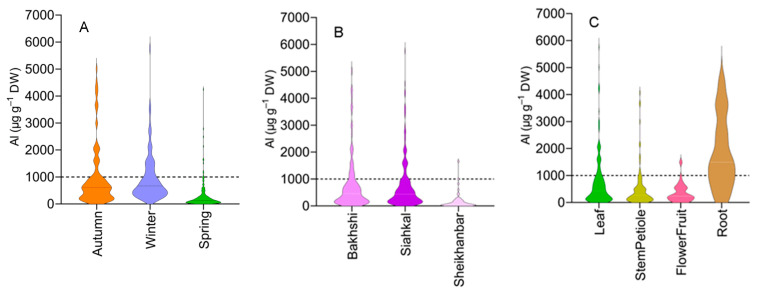
Presentation of the effect of collection season (**A**), location (**B**) and plant organ (**C**) on the Al concentration of the plant specimens growing on acid soils in Northern Iran.

The Al concentration data sorted by location, collection time, and plant organ (Figure 1) revealed that most specimens with Al concentrations greater than 1000 µg g^−1^ DW belonged to collections made in autumn and winter. These specimens also showed the highest maximum level of Al as compared to the specimens collected in spring (Figure 1A). The maximum Al concentration was greater in the tea garden samples (‘Bakhshi’ and ‘Siahkal’) than in the surrounding area samples (‘Sheikhanbar’) (Figure 1B). Al concentrations greater than 1000 µg g^−1^ DW were predominantly observed in the roots, followed by the leaves, stem, and petioles. Flowers and fruits had the lowest maximum Al concentration among the specimens examined (Figure 1C).

**Figure 2:**
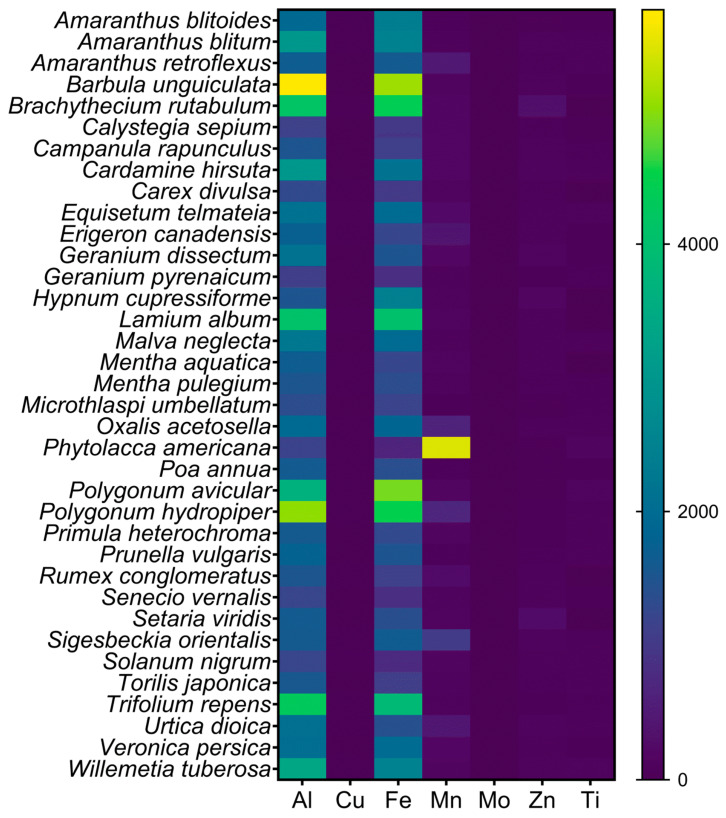
Heat map presenting the shoot concentrations (µg g^−1^ DW) of Al and micronutrients and Ti of Al-accumulating species.

Al concentrations greater than 1000 µg g^−1^ DW were detected in the shoots of 36 species from 23 families (Table 1). In the aerial parts of these species, an average concentration of 2223 µg Al g^−1^ DW was found. Maximum Al accumulation was observed in the leafy shoots of the bryophyte *Barbula unguiculata*, which contained up to 5752 µg Al g^−1^ DW. Fe and Mn levels were also relatively high in these species. *B. unguiculata* had the highest Fe accumulation (5094 µg g^−1^ DW) among the analyzed species, while *Phytolacca americana* had the highest Mn concentration in the leaves (5454 µg Mn g^−1^ DW) (Figure 2).

Due to phenological differences, most autumn- and winter-collected species were absent in spring, and vice versa. Only 17 species (out of a total of 89 species) were present in all three collection seasons, and only four of these exhibited a consistent Al accumulation trait, regardless of the season; in the remaining species, this trait was only observed in autumn and winter specimens (Table S2).

The species with shoot Al concentrations below 1000 µg g^−1^ DW, regardless of location or time of collection, were designated as Al excluders (non-accumulators). This group included 50 species (Figure S1). In this group, *Hypericum perforatum* L. (Hypericaceae) had the highest Al leaf concentration with approximately 992 µg Al g^−1^ DW, followed by *Potentilla reptans* L. (Rosaceae) (978 µg g^−1^ DW) and *Sambucus ebulus* L. (Rosaceae) (947 µg g^−1^ DW). The tree species *Populus deltoides* had the lowest Al concentration, with 17–28 µg Al g^−1^ DW in the young leaves and 45–62 µg Al g^−1^ DW in the old leaves. The herbaceous species *Agrimonia eupatoria* (Rosaceae) had the second-lowest Al concentration, with 177 µg Al g^−1^ DW in the leaves.

The highest leaf concentration of nonessential trace elements (Cd, Co, Cr and Pb) and Ni (micronutrient) referred to here as heavy metals (HM) was observed for Cr, followed by Ni for both accumulator and excluder species (Figure S2).

In addition to the difference in Al concentration between accumulator and excluder species (*p* < 0.001), there was also a significant difference in the concentration of micronutrients (Cu, Fe, Mn, Mo, Zn and B) between these species (Table S3). Except for Cd, the accumulating species contained significantly more Co, Cr, Ni and Pb than the excluder species. However, macronutrient concentrations (except Ca) did not differ significantly between the two groups (Table S3).

**Figure 3:**
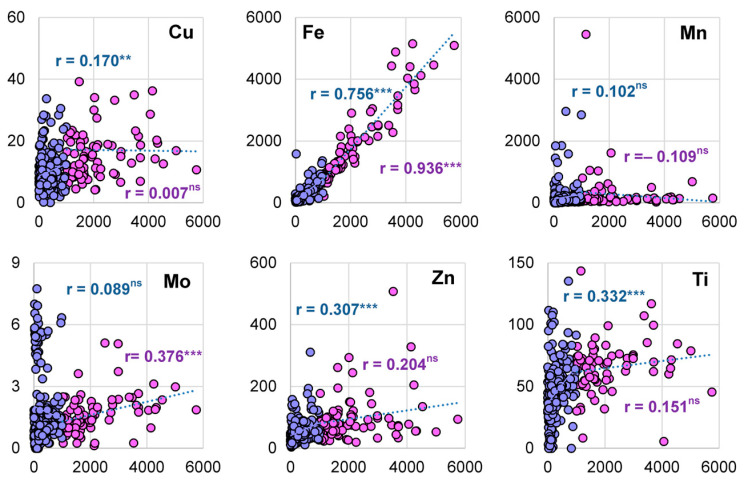
The correlation (Pearson’s correlation coefficient) between the concentrations of Al with that of micronutrients and Ti in the Al accumulator (magenta) and excluder (blue) species. Significant correlations were indicated by the asterisks. *** *p* < 0.001, ** *p* < 0.01, ns, non-significant.

In Al-accumulating species, a significant positive correlation was found between Al concentration in specimens with Fe and Mo concentrations but not with Cu, Mn and Zn (Figure 3). The concentration of Al in these species was also found to be positively correlated with Co, Cr, Ni and Pb (Figure S3).

Similar to Al accumulators, a positive correlation was observed between Al and Fe in the excluder species. In contrast to accumulating species, however, a significant correlation was observed between Al and Cu and Zn, but not between Al and Mo (Figure 3). Among HMs, only Co concentration was correlated with Al concentration in the excluder species (Figure S3).

Ti was also analyzed in the specimens in order to estimate the probable contribution of dust and soil contamination to the Al accumulation data. The concentration of Ti in the specimens ranged from 0–143 µg g^−1^ DW. Only one specimen with a high Ti concentration (1040 µg g^−1^ DW) and high Al, Fe and Mn levels was found and removed from the data list. Although the Ti concentration in the Al-accumulating species was significantly higher than that in the excluder species (Table S3), no correlation was observed between Al and Ti in the accumulating species (Figure 3).

**Figure 4:**
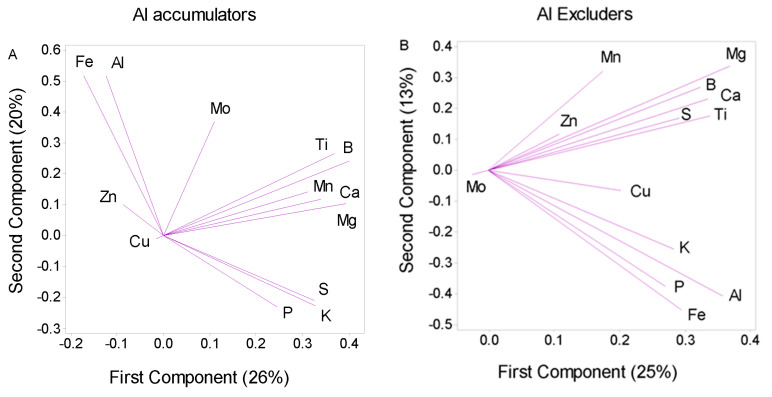
The unconstrained ordination (PCA) of shoot concentration data of Al, macro- and micronutrients and Ti in the plant specimens belonging to Al accumulators ((**A**), 82 specimens) and non-accumulators ((**B**), 364 specimens) growing on acid soils in Northern Iran. The share of total variance represented by each PCA component is shown in parentheses.

The PCA analysis of shoot elemental concentrations revealed a distinct difference between Al accumulators and excluders. A close association was found between Al and Fe in the Al accumulators. At the same time, other elements, such as P, K and S, on one side, and Ca, Mg, Mn and B, were grouped in different clusters (Figure 4A). By contrast, in the excluder species, all elements were clustered together except for Mo, which was clustered separately, similar to the accumulators (Figure 4B). The clustering of Ti in a different group than Al and Fe in the accumulators confirmed that soil contamination had a negligible effect on Al accumulation in our plant samples.

### 2.2. Accumulation of Al in Plants under Laboratory Conditions

**Figure 5:**
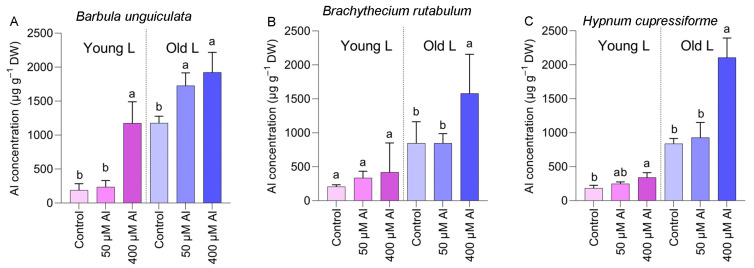
Al accumulation in the young and old leaves of three bryophyte species, *Barbula unguiculata* (**A**), *Brachythecium rutabulum* (**B**) and *Hypnum cupressiforme* (**C**) treated for four weeks with three Al concentrations. Significant differences among three Al treatments (within each leaf type) were indicated by different lower case letters (*p* < 0.05).

All three bryophyte species exhibit a high capacity for Al accumulation, especially in the older leaves (Figure 5). Since the parental plants were collected from tree barks, it was not expected that the culture experiment without Al treatment would result in a high Al accumulation. However, in *B. unguiculata*, a high Al accumulation (1177 µg g^−1^ DW) was found in the old leaves, even cultivated in the absence of Al (Figure 5A). *H. cupressiforme* had the highest Al accumulation among hydroponically grown plants, with old leaves containing up to 2500 µg Al g^−1^ DW (Figure 5C).

**Figure 6:**
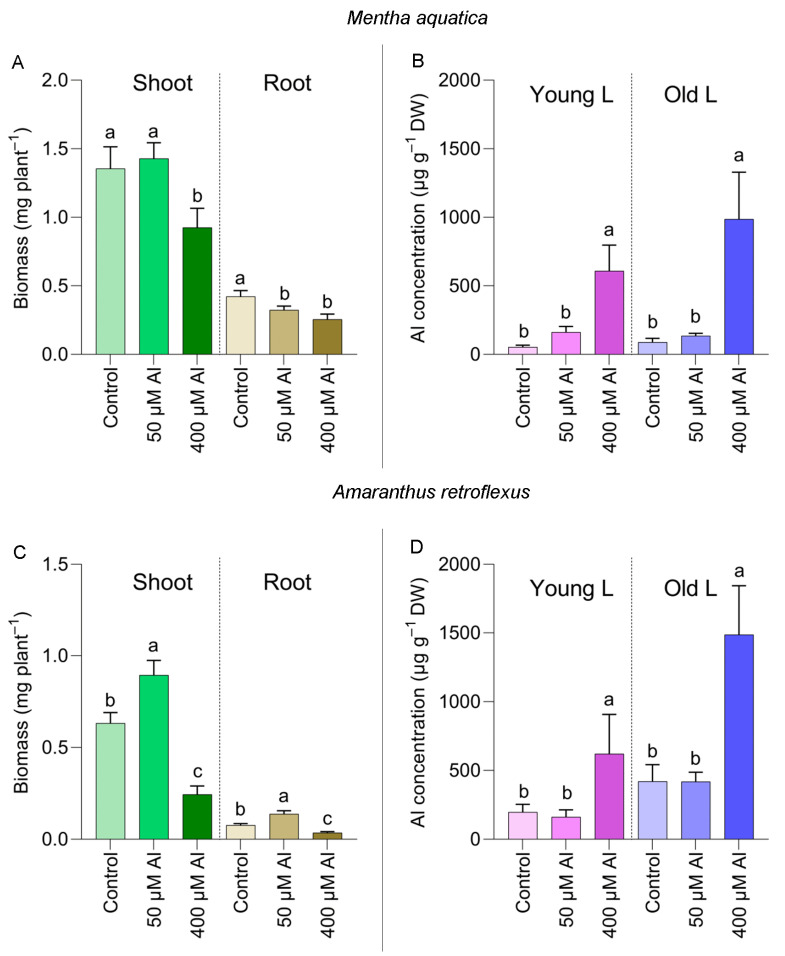
Biomass and Al accumulation in the young and old leaves of *Mentha aquatica* (**A**,**B**) and *Amaranthus retroflexus* (**C**,**D**) grown for four weeks under three Al concentrations in the nutrient solution. Significant differences among three Al treatments (within each leaf type) were indicated by different lower case letters (*p* < 0.05).

The shoot biomass of *M. aquatica* was unaffected by 50 µM Al but decreased by approximately 32% when 400 µM Al was present (Figure 6A). However, a reduction in root biomass was also detected under 50 µM Al treatment (Figure 6A). Only 400 µM Al treatment resulted in a significant increase in Al concentration in the leaf. On the other hand, Al accumulations greater than 1000 µg g^−1^ DW were observed only in the older leaves (Figure 6B).

The shoot and root biomass of *A. retroflexus* was significantly improved under 50 µM Al but decreased by 400 µM Al (Figure 6C). Al accumulation in this species reached 1898 µg g^−1^ DW in older leaves of plants treated with 400 µM Al for four weeks (Figure 6D).

## 3. Discussion

### 3.1. Al Accumulation in Species Not Previously Recorded as Al Accumulators

According to our knowledge, none of the plant species identified in this study as Al accumulators have previously been reported for this trait. This is not surprising given that most research on Al-accumulating species has focused on plant species inhabiting acid soils of the two geographic belts in the boreal and tropical regions. Although the geographic distribution patterns of plant species are governed by a complex set of variables, including edaphic factors, climatic factors are the most significant predictors of phytogeographic patterns [19]. The acid soils formed occasionally in other biomes under different climatic conditions are inhabited by different species adapted to local climatic conditions along with soil acidity. The study areas in our work have been devoted to tea cultivation since the beginning of the 20th century and are located in the foothills of the northern slopes of the Alborz Mountains belonging to the Hyrcanian forests stretch. This area is a diverse hot spot in southwest Asia [20] with unique flora and vegetation [21] and was designated as a UNESCO World Heritage property (https://whc.unesco.org/en/list/1584, accessed on 26 May 2023).

The plants analyzed in this study have not been reported as Al accumulators at the level of genus (except *Setaria* and *Rumex*) or family (except Poaceae and Polygonaceae) in the list of accumulators reviewed by Jansen et al. [9,22] and subsequently published works. This again emphasizes the uniqueness of the acid soils in Northern Iran, which support a unique flora distinct from the flora of other acid soils already explored by plant biologists worldwide. This suggests that for a comprehensive list of Al accumulators, surveys are required of other floristic units formed in each acid soil in the world.

Most of the 86 species collected and analyzed (50 species, or 60% of the total) did not exhibit the Al accumulation trait and are classified as excluder species. This suggests that exclusion may be an equally or even more effective strategy for confronting Al toxicity in acid soils. In-depth research on these species could lead to the discovery of new model organisms and expand our understanding of the Al exclusion mechanisms in plants.

### 3.2. Accumulation of Al in Different Seasons and Locations

Only a small proportion of the collected species (19%) were present in all three seasons, and approximately 76% of these species showed the Al accumulation trait only in autumn and winter specimens. This could be due to a longer growth period allowing plants to accumulate Al, particularly in mature leaves with a longer life span. Mature leaves are typically richer in biochemical compounds than younger leaves [6], which may play a role in Al chelation and detoxification.

The Al accumulation range in the collected specimens is lower, with a median of 1689 µg g^−1^ DW and mean (±SD) of 2223 ± 1179, than other reports on accumulators in the tropics, which show Al accumulation primarily in the range of 4310–>10,000 µg g^−1^ DW, but much higher levels have also been recorded, for example, 30,500 µg g^−1^ DW Al observed in *Memecylon laurinum* [22]. This could be due to the higher inherent capacity of tropical species for Al accumulation. Leaf longevity could also play a role. The identified perennial species have deciduous leaves, and 33% (12 of 36) of accumulator species are annual plants, while the high Al concentration reported for tree species in the tropics is the result of an unknown active period of uptake and accumulation.

To the best of our knowledge, the Al accumulation capacity has been evaluated based solely on leaf concentration data, while the Al uptake and accumulation within a given time frame have not been specifically examined in these species. The accumulation of Al (>1000 µg g^−1^ DW) in the old leaves of *A. retroflexus* and *M. aquatica* grown for only four weeks at 400 µM Al (Figure 6) suggests a remarkable Al accumulation capacity in these species. A higher Al accumulation in the bryophytes examined in this study is also likely related to the longevity of their fronds. All 36 Al-accumulating species are herbaceous, whereas all 12 tree and woody species collected and analyzed are Al excluder species. This is likely because soil leaching, the primary cause of soil acidification in the study area, leads mainly to topsoil acidity, and it is the topsoil that is the primary site of the roots of annual herbaceous species. The opposite is the case for tropical soils with subsoil acidity, characterized by a predominance of woody species with Al accumulation [10].

### 3.3. Accumulation of Al and Concentration of Micronutrients

High Fe^2+^ availability in acid soils, which can lead to Fe toxicity in susceptible species, is another challenge for plants growing in these conditions [23]. Similar to other elements with an excess concentration in soil, plants in acid soils may employ exclusion or accumulation/internal detoxification strategies to combat excess Fe availability [24]. For the Al-accumulating plants in this study, a high Fe concentration (range: 1026–5155 µg g^−1^ DW; median: 1467) was observed in the leaves of all species, exceeding the toxicity threshold of 500 µg g^−1^ DW [25]. In addition, the PCA (Figure 4) and Pearson’s correlation (Figure 3) analyses support the co-accumulation of Al and Fe in the accumulating species.

The Al excluders had significantly lower leaf Fe concentrations (range: 21–935 µg g^−1^ DW; median: 203) than the Al accumulators (*p* < 0.001; Table S3). Nevertheless, PCA (Figure 4) and Pearson’s correlation (Figure 3) analysis revealed a positive relationship between Al and Fe concentration in these species similar to that observed in the accumulators. This may indicate a link between the two elements in both accumulators and excluders. Al is likely co-precipitated with ferric compounds in the leaves [26], resulting in decreased levels of free Fe^2+^ and Al^3+^ ions in the cytosol. In agreement with this assumption, we observed an increase in Fe demand in both accumulating and excluding species when they were exposed to low Al concentrations (50 µM). These plants exhibit leaf chlorosis unless supplemented with additional Fe (unpublished data). To this end, hydroponically grown tea plants exhibit Fe toxicity in the absence of Al [24]. Thus, the parallel accumulation of Fe and Al, as indicated by the positive correlation between tissue concentrations of these elements, is likely an adaptive mechanism for compensating the Al-induced Fe inactivation in the leaves of both accumulators and excluders.

Although Mn availability is also increased under acid soil conditions [27], Al and Mn did not co-accumulate, as confirmed by the absence of a correlation between their concentrations in accumulator and excluder species (Figure 3). This highlights the specific relationship between Al and Fe in plants adapted to acid soils.

Although Mo availability is low in acid soils due to the strong adsorption of the molybdate anion (MoO_4_^2−^) on the surface of Fe- and Al-oxides [28], the Mo concentration was positively correlated with Al in the accumulator but not in the excluder species. This may indicate an additional adaptation of Al-accumulating species that warrants further study in these and other Al accumulators native to acid soils.

Despite the absence of a report on Al accumulation, some of these species have already been documented as possessing the HM accumulation trait (Table S4). Heavy metal hyperaccumulation usually is metal specific or involves chemically similar elements co-occurring at the contaminated site, such as Zn and Cd [29,30]. An association has been found among accumulation traits for different metals by Broadley et al. [31], although Al was not included in this study. Hyperaccumulation of both Al and HMs has been revealed in a phylogenetic study of the Plantaginaceae family [32]. A significantly higher HM accumulation (except Cd) in the Al accumulators in our study also suggests that the physiological and genetic requirement for HM accumulation and tolerance is partly related to that of Al. Thus, these species could all be a candidate for HM accumulation studies and likely contain some potent HM hyperaccumulators.

### 3.4. Accumulation of Al from an Ecological and Evolutionary Point of View

The criteria set for the definition of accumulation and hyperaccumulation of HMs and Al are a matter of discussion among plant biologists [33,34]. The introduced list of Al-accumulating species in this study is consistent even with the exacting definition of hyperaccumulation given by van der Ent et al. [35] since the data have been derived from plants in natural habitat, found in the leaves of at least one specimen of a given species (two specimens in our work, each consisted from 2–4 individual plants), proved to be free from air-borne deposition of dust and was also confirmed (for selected species) under standard cultural conditions. Moreover, leaf Ti concentrations were analyzed as an indicator of soil contamination of the samples.

The distribution of the Al accumulation trait among angiosperms has been studied to determine the evolutionary basis for this trait. When plotted on the classification systems of several orders of angiosperms, the scattered distribution of Al hyperaccumulation led some taxonomists to conclude that Al hyperaccumulation has no taxonomic value, particularly at higher levels [36]. Conversely, other scientists deemed the current data on Al-accumulating species insufficient and advocated examining a broader range of taxa to investigate Al hyperaccumulation’s origin and taxonomic significance [9]. The application of molecular phylogenies to the known accumulators led to the conclusion that Al accumulation is a primitive trait that has arisen independently multiple times but is likely lost in the most derived taxa [9]. Our data may contribute to expanding the number of Al accumulators at the species, genus and family levels and facilitate phylogenetic analyses of the distribution of hyperaccumulation throughout the plant kingdom.

Of the 36 Al accumulators discovered, 13 are cosmopolitan, and 10 are pluriregional, implying that these species (64% of all found Al accumulators) colonized the acid soils due to favorable climatic conditions such as mild temperature and high moisture. Two main hypotheses explain the evolution of hyperaccumulation. Some researchers propose that hyperaccumulation features are caused by genetic drift, indirect selection and their combinations [37]. On the other hand, other scientists believe that this trait evolved as a consequence of natural selection in plants growing in metalliferous soils millions of years ago [33]; accumulator species are thus expected to have a high rate of endemism [35]. Indeed, a localized geographic distribution of HM-rich soils has led to edaphic endemism for HM tolerance and accumulation traits. Heavy metal hyperaccumulation has independently evolved many times at the subgenus level, frequently as subspecies or varieties [38]. By contrast, high soil Al is not localized but has been distributed widely in both hemispheres leading to the evolution of Al accumulation in larger taxonomic groups, i.e., closely related genera, subfamilies or families or tribes [9]. The lack of endemism in the majority of the discovered Al-accumulating species in this study may imply that Al accumulation is a widely distributed trait in angiosperms and lower plants. This feature appears to be latent in most of their habitats but is occasionally visible in acid soil conditions.

The remaining 13 species belong to the Euro-Siberian phytogeographic region exclusively (2 species) or in common with other units: the Hyrcanian and Euxino-Hyrcanian (5 species), the Irano-Turanian (3 species) or the Mediterranean (3 species). The presence of a center of diversification for Al accumulation in this phytogeographic region may be suggested by the high frequency of Euro-Siberian elements (36%) in our collected Al-accumulating species. Indeed, the distribution of acid soils in the northern belt overlaps substantially with the Euro-Siberian (boreal) phytogeographic region.

## 4. Materials and Methods

### 4.1. Study Area

The study area is situated in Northern Iran (Guilan Province), in the western part of the south Caspian region, with an area of approximately 200 km^2^ lying between 37°10′–37°30′ northern latitude and 49°80′–50°10′ eastern longitude (Figure S4), with an altitude of 0–54 m, an annual temperature of 16.1 °C and annual precipitation of 1455.2 mm. The bioclimatic classification for this region is temperate oceanic [39]. Plant and soil samples were collected from two primary areas: ‘Bahkshi’ (a private tea garden) and ‘Siahkal’ (a tea garden under the supervision of the Tea Research Center, AREEO). The areas around these locations (‘Sheikhanbar’) were also visited to collect plant and soil samples.

### 4.2. Collection of Soil Samples

The soil samples were collected from 0–25 cm deep to assess the physical and chemical properties shown in Tables S5 and S6. The soils in the study area are classified as Inceptisols and Entisols with udic and thermic moisture and temperature regimes, respectively [40].

### 4.3. Collection of Plants and Determination of Species

Plants were collected in autumn (2018), winter, and spring in succession (2019, 2020). The collected vegetation was labeled, placed in plastic bags and transported to the laboratory. Using plant identification references, plants were identified immediately in the field or after transfer to the laboratory and compared with specimens from the Herbarium of the Agricultural and Natural Resources Research Centre of Guilan (GILAN) [41,42].

### 4.4. Preparation of Samples and Elemental Analyses

The collected plants were washed for 10 min under running tap water to remove dust and soil particles from their surfaces. The roots and shoot fractions (young and old leaves, stem, petiole, flower and fruits, if applicable) were then separated, washed twice more for 15 min with distilled water and blotted dry with filter paper. The samples of distinct fractions derived from two to four individual plants of a given species were combined, labeled and considered a specimen; a total of 499 specimens were prepared and subjected to the elemental analyses described below. For each fraction, at least two specimens (within each location and time) were prepared. The plants from each collection location and time were prepared and labeled separately. The samples were oven-dried for 48 h at 70 °C, milled and sent to the Institute for Multidisciplinary Research, University of Belgrade, Serbia, for elemental analyses as described below.

The samples were microwave digested in 3 mL of concentrated HNO_3_ and 2 mL of H_2_O_2_ for 1 h (ETHOS EASY, Milestone Srl, Sorisole, Italy), then transferred into 25 mL plastic flasks, and the volume was adjusted to 25 mL with deionized water. Element concentrations were determined using inductively coupled plasma optical emission spectroscopy (ICP-OES; Spectro-Genesis EOP II, Spectro Analytical Instruments GmbH, Kleve, Germany). Titanium was included in the elemental analysis to serve as a possible indicator of soil contamination in environmental samples [43]. The certified reference material (GBW10015 Spinach; Institute for Geophysical and Geochemical Exploration, Langfang, China) was used to evaluate the precision and accuracy of the analyses.

### 4.5. Plants Cultivation and Treatment under Growth Chamber Conditions

For the study of Al tolerance and accumulation capacity, five species (including three bryophytes) were hydroponically cultivated in a growth chamber under the conditions outlined below. A low-strength Hoagland nutrient solution (pH 4.0) was used to obtain a high Al activity in the medium. GEOCHEM-PC was utilized to calculate the free Al^3+^ activity and its speciation in the solution (Table S7).

Three levels of Al treatments, including 0, 50, and 400 µM Al (as AlCl_3_), with free Al^3+^ activities of 0, 11.97 µM and 125 µM, respectively, were applied to the plants. The plants were grown in a growth chamber with a photoperiod of 16/8h day/night, a day/night temperature range of 27/16 °C, and relative humidity of 70–80%. The light was provided by fluorescent lamps with a photon flux density of approximately 300 μmol m^−2^ s^−1^.

The bryophyte species’ (*Barbula unguiculata*, *Brachythecium rutabulum*, *Hypnum cupressiforme*) leafy shoots were carefully removed from the tree barks, placed in plastic bags, sprayed with distilled water to keep the plants moist, and then transferred to the laboratory. The shoots were washed with distilled water and placed on a thin sponge pad within 20 cm L × 12 cm W × 10 cm H transparent polypyrene containers; the lids were kept closed to prevent additional evaporation. The plants were irrigated with a nutrient solution that was applied directly on the sponge pads. The volume of nutrient solution applied for each container was 200 mL per week. After four weeks of Al treatments, the plants were harvested. After washing with distilled water for 10 min, the young and old leaves were separated and prepared for Al analyses as described above.

Seeds of *Amaranthus retroflexus* were collected from a tea garden within the study area. After surface-sterilizing with 10% sodium hypochlorite, the seeds were germinated in perlite in the dark. Young seedlings were transferred to light and irrigated for an additional week with a 25% Hoagland nutrient solution. Similar two-week-old plants were precultured in hydroponics (pH 6.0). Two weeks after the preculture, the nutrient solution was replaced with the low-strength nutrient solution (pH 4.0) described above, and weekly replacements were performed. Plants were harvested four weeks after the start of Al treatments, and in addition to the biomass, the young leaves, old leaves and roots were used for Al analyses.

Whole *Mentha aquatica* plants were collected from a tea garden in the study area and transferred to the laboratory. Young shoot cuttings with four developed leaves and two internodes were transferred to the Hoagland solution for rooting following acclimatization to growth chamber conditions and hydroponic medium. Ten days after plants were transferred to this medium, they were transferred to treatment pots, and Al treatments were initiated. Nutrient solutions were exchanged weekly. Plants were harvested four weeks after starting Al treatments for biomass and Al accumulation.

### 4.6. Data Analysis of Field Samples and Design of the Lab Experiments

After the ICP analyses of the field specimens were completed, data were labeled with the respective species name, organ, collection area and season. The data were then sorted for Al concentration, and specimens with Al greater than 1000 µg g^−1^ DW were marked separately for each location and collection season. Following the exclusion of species with only root Al accumulation, plant species with Al accumulation in the aerial parts (with or without root accumulation) in at least two specimens (each comprising 2–4 individual plants, thus, in at least 4 individual plants) collected from the same or different locations and seasons, were designated as Al accumulator species( Table 1).

Minitab 18 was used for statistical analyses such as Principle Component Analysis (PCA), analysis of differences in element concentrations in accumulators and excluders (Mann–Whitney test) and correlation of Al concentration with other elements within each group (Pearson’s correlation coefficient). For PCA, the cross-product matrix of correlation coefficients was used.

Plant cultivation under growth chamber conditions was performed using a completely randomized design with four independent containers or pots, i.e., four replicates per treatment. The pairwise comparison of means was performed with Tukey’s test (*p* < 0.05) using Sigma Stat 3.02. GraphPad Prism 9.0 was used to create the graphs.

## 5. Conclusions

To date, most identified Al accumulators are tree and woody species from the two geographic belts in the northern and southern hemispheres, with little known about accumulators from other diversification and phytogeographic regions. In this study, we identified 36 species as Al accumulators that have not previously been reported for this trait. In addition to their phylogenetic and evolutionary significance, these accumulator species could be used as new models for the study of Al hyperaccumulator physiology and for exploring novel internal and external detoxification mechanisms. Additionally, some cosmopolitan species, e.g., *Amaranthus* spp., may be beneficial in the rehabilitation and revegetation of acid-eroded soils all over the world. Some species that are closely related to the agronomic species, e.g., *Solanum nigrum* and *Trifolium repens* (Al accumulators) and *Solanum pseudocapsicum* and *Vicia tetrasperma* (Al excluders), could also be used for developing Al-tolerant cultivars for the related crop species.

## Figures and Tables

**Table 1:** The list of plants (consisting of 36 species belonging to 23 families) growing in acid soils in Northern Iran with shoot Al concentrations higher than 1000 µg g^−1^ DW of at least four individual plants and the range of Al concentration.

Species	Family	Al Concentration (µg g^−1^ DW)
*Amaranthus blitoides* S. Watson	Amaranthaceae	1930–2992
*Amaranthus blitum* L.	Amaranthaceae	1578–2672
*Amaranthus retroflexus* L.	Amaranthaceae	1108–1655
*Barbula unguiculata* Hedw.	Pottiaceae	2132–5752
*Brachythecium rutabulum* (Hedw.) Schimp.	Brachytheciaceae	1180–1292
*Calystegia sepium* (L.) R. Br.	Convolvulaceae	1120–1139
*Campanula rapunculus* L. subsp. *lambertiana* (A.DC.) Rech. f.	Campanulaceae	1209–1495
*Cardamine hirsuta* L.	Brassicaceae	1570–2981
*Carex divulsa* Stokes	Cyperaceae	1010–1295
*Equisetum telmateia* Ehrh.	Equisetaceae	1183–2114
*Erigeron canadensis* L.	Asteraceae	1023–2028
*Geranium dissectum* L.	Geraniaceae	2072–2153
*Geranium pyrenaicum* Burm. f.	Geraniaceae	1005–1101
*Hypnum cupressiforme* Hedw.	Hypnaceae	1102–1501
*Lamium album* L.	Lamiaceae	2151–4061
*Malva neglecta* Wallr.	Malvaceae	1635–2237
*Mentha aquatica* L.	Lamiaceae	1654–5336
*Mentha pulegium* L.	Lamiaceae	1034–1511
*Microthlaspi umbellatum* F. K. Mey.	Brassicaceae	1011–1342
*Oxalis acetosella* L.	Oxalidaceae	1420–1954
*Phytolacca americana* L.	Phytolaccaceae	1098–1156
*Poa annua* L.	Poaceae	1615–3700
*Polygonum aviculare* L.	Polygonaceae	1578–3627
*Polygonum hydropiper* L.	Polygonaceae	1030–5008
*Primula heterochroma* Stapf	Primulaceae	1250–1581
*Prunella vulgaris* L.	Lamiaceae	1023–1782
*Rumex conglomeratus* Murray	Polygonaceae	1311–1522
*Senecio vernalis* Waldst. & Kit.	Asteraceae	1159–1209
*Setaria viridis* (L.) P. Beauv.	Poaceae	1240–1618
*Sigesbeckia orientalis* L.	Asteraceae	1086–1601
*Solanum nigrum* L.	Solanaceae	1143–1208
*Torilis japonica* (Huott.) DC.	Apiaceae	1076–1412
*Trifolium repens* L.	Fabaceae	3707–4299
*Urtica dioica* L.	Urticaceae	1007–2095
*Veronica persica* Poir.	Plantaginaceae	1194–2044
*Willemetia tuberosa* Fisch. & C. A. Mey. Ex DC.	Asteraceae	1456–3376

## Data Availability

The data presented in this study are available upon request from the corresponding author.

## Supplementary Materials

The following supporting information can be downloaded at: https://www.mdpi.com/article/10.3390/plants12112129/s1. Figure S1: Heat map presenting the concentration (µg g^−1^ DW) of Al, micronutrients and Ti in the aerial parts (shoots) of excluder species; Figure S2: Heat map presenting the concentration (µg g^−1^ DW) of heavy metals in the aerial parts (shoots) of Al-accumulating (above panel) and Al-excluding (below panel) species; Figure S3: The correlation (Pearson’s coefficient) between the concentration of Al with that of heavy metals and Se in the Al-accumulating (magenta) and -excluding (blue) species; Figure S4: Study area in the south of the Caspian Sea, Northern Iran (A) and the collection sites (B and C, tea gardens; D and E, surrounding area); Table S1: The list of collected and analyzed plant species from acid soils in Northern Iran. The list consists of 86 species belonging to 43 families including 36 Al-accumulating species (designated in bold) from 23 families; Table S2: List of plant species collected in all three seasons from acid soils in Northern Iran; Table S3: The difference (*p*-values, Mann–Whitney test) between Al accumulators and Al excluders in the concentrations of micronutrients, macronutrients, heavy metals and Se in plant specimens collected from acid soils in Northern Iran; Table S4: The accumulation feature for heavy metals (HM) and Al for the species designated as Al accumulator in this study [44,45,46,47,48,49,50,51,52,53,54,55,56,57,58,59,60,61,62,63,64]; Table S5: Physical and chemical properties and the available and total concentration of mineral elements in the soil samples collected from three locations in the study area [65]; Table S6: Soil chemical properties in the samples collected in three successive years and three seasons from the Siahkal Tea Research Station; Table S7: The composition of nutrient solution (pH 4.0) and the Al concentration and the free Al^3+^ activity (calculated using GEOCHEM-PC) used for hydroponic culture of plants.
