# Supplementary material for: Weed Species from Tea Gardens as a Source of Novel Aluminum Hyperaccumulators
Source: Plants (Basel). 2023 May 27;12(11):2129. doi: 10.3390/plants12112129 (PMC10255622; doi:10.3390/plants12112129)
Supplement: Supplementary file 1 [file plants-12-02129-s001.zip › plants-2327710-PublishRevise-supplementary-5.30-1.pdf]

Table S1. The list of collected and analyzed plant species from acid soils in North Iran. The list consists of 86 species belonging to 43 families including 36 Al accumulating species (designated in bold) from 23 families.

Family	Species	Life form*	Habit**	Chorotype***
Adoxaceae	*Sambucus ebulus* L.	Ge	P–Herb	ES, M
Amaranthaceae	***Amaranthus blitoides* S.Watson**	**Th**	**A–Herb**	**Cosm**
	***Amaranthus blitum* L.**	**Th**	**A–Herb**	**Cosm**
	***Amaranthus retroflexus* L.**	**Th**	**A–Herb**	**Cosm**
	Chenopodium album L.	Th	A–Herb	Cosm
Apiaceae	*Froriepia subpinnata* (Ledeb.) Baill.	He	P–Herb	ES (Hyr)
	Pimpinella affinis Ledeb.	He	P–Herb	IT
	***Torilis japonica* (Huott.) DC.**	**Th**	**A–Herb**	**ES–IT**
Araliaceae	*Hedera pastuchovii* Woronow	Ph	P–Woody-Climber	ES (EH)
Aspleniaceae	*Asplenium adiantum-nigrum* L.	Ge	P–Herb	Pl
Asteraceae	*Artemisia annua* L.	Th	A–Herb	ES, IT
	Artemisia vulgaris L.	He	P–Herb	ES, IT
	Dichrocephala integrifolia (L.f.) Kuntze	Th	A–Herb	Pl
	Erigeron annuus (L.) Pers.	Th	A–Herb	Naturalized
	***Erigeron canadensis* L.**	**Th**	**A–Herb**	**Cosm**
	Lapsana communis L.	He	P–Herb	Pl
	***Senecio vernalis* Waldst. & Kit.**	**Th**	**A–Herb**	**ES (EH)**
	***Sigesbeckia orientalis* L.**	**Th**	**A–Herb**	**Pl**
	Urospermum picroides (L.) Scop. Ex F.W. Schmidt	Th	A–Herb	Pl
	***Willemetia tuberosa* Fisch. & C. A. Mey. Ex DC.**	**Ge**	**P–Herb**	**ES (Hyr)**
Betulaceae	*Alnus subcordata* C. A. Mey.	Ph	Tree	ES (Hyr)
Brachytheciaceae	***Brachythecium rutabulum* (Hedw.) Schimp.**	**He-Thallo-Ep**	**Br–P**	**ES, M**
Brassicaceae	***Cardamine hirsuta* L.**	**Th**	**A–Herb**	**Cosm**
	***Microthlaspi umbellatum* F. K. Mey.**	**Th**	**A–Herb**	**ES (EH)**
	Rapistrum rugosum (L.)All.	Th	A–Herb	E, IT, M
	Sisymbrium officinale (L.)Scop.	Th	A–Herb	Cosm
Buxaceae	*Buxus sempervirens* L. subsp. Hyrcana (Pojark.) Takht.	Ph	Tree	ES (Hyr)
Campanulaceae	***Campanula rapunculus* L. subsp. *lambertiana* (A. DC.) Rech. f.**	**He**	**P–Herb**	**ES (EH), IT**
Caryophyllaceae	*Cerastium glomeratum* Thuill.	Th	A–Herb	Cosm
	Stellaria media (L.) Vill.	Th	A–Herb	Cosm
Commelinaceae	*Commelina communis* L.	He	P–Herb	Naturalized

	Tradescantia fluminensis Vell.	He	P–Herb	Naturalized
Convolvulaceae	***Calystegia sepium* (L.) R.Br.**	**Ge**	**P–Herb**	**Pl**
Cyperaceae	***Carex divulsa* Stokes**	**He**	**P–Sedge**	**ES, M**
Dennstaedtiaceae	*Pteridium aquilinum* (L.) Kuhn	Ge	P	Cosm
Equisetaceae	***Equisetum telmateia* Ehrh.**	**Ge**	**P**	**Pl**
Euphorbiaceae	*Acalypha australis* L.	Th	A–Herb	Cosm
	Euphorbia maculata L.	Th	A–Herb	Cosm
Fabaceae	*Albizia julibrissin* Durazz.	Ph	Tree	ES (EH) [China/Japan]
	Gleditsia capsica Desf.	Ph	Tree	ES (Hyr)
	***Trifolium repens* L.**	**He**	**P–Herb**	**Pl**
	Vicia tetrasperma (L.) Schreb.	Th	A–Herb	ES, IT, M
Fagaceae	*Quercus castaneifolia* C. A. Mey.	Ph	Tree	ES (Hyr)
Gentianaceae	*Centaurium erythraea* Rafn	Th	A–Herb	Pl
Geraniaceae	***Geranium dissectum* L.**	**Th**	**A–Herb**	**Pl**
	***Geranium pyrenaicum* Burm. f.**	**He**	**P–Herb**	**Cosm**
Hypericaceae	*Hypericum perforatum* L.	He	P–Herb	Cosm
	Hypericum tetrapterum Fr.	He	P–Herb	ES–M
Hypnaceae	***Hypnum cupressiforme* Hedw.**	**He-Thallo-Ep**	**Br-P**	**Cosm**
Juncaceae	*Juncus effusus* L.	Ge	P–Sedge	Pl
Lamiaceae	***Lamium album* L.**	**He**	**P–Herb**	**Pl**
	***Mentha aquatica* L.**	**He**	**P–Herb**	**ES**
	***Mentha pulegium* L.**	**He**	**P–Herb**	**ES**
	***Prunella vulgaris* L.**	**He**	**P–Herb**	**Pl**
	Teucrium hircanicum L.	He	P–Herb	ES (Hyr)
Lythraceae	*Lythrum salicaria* L.	He	P–Herb	Pl
Malvaceae	***Malva neglecta* Wallr.**	**He**	**P–Herb**	**ES, IT, M**
Onagraceae	*Epilobium hirsutum* L.	He	P–Herb	Pl
Oxalidaceae	***Oxalis acetosella* L.**	**He**	**P–Herb**	**Pl**
Phytolaccaceae	***Phytolacca americana* L.**	**He**	**P–Herb**	**Cosm**
Plantaginaceae	***Veronica persica* Poir.**	**He**	**P–Herb**	**Cosm**
Poaceae	*Alopecurus myosuroides* Huds.	Th	A–Grass	Pl
	Cynodon dactylon L.	He	P–Grass	Pl
	Digitaria sanguinalis (L.) Scop.	Th	A–Grass	Pl
	Paspalum dilatatum Poir.	Ge	P–Grass	Cosm
	Paspalum distichum L.	He	P–Grass	Cosm
	***Poa annua* L.**	**Th**	**A–Grass**	**Cosm**
	***Setaria viridis* (L.) P. Beauv.**	**Th**	**A–Grass**	**Pl**
Polygonaceae	***Polygonum aviculare* L.**	**Th**	**A–Herb**	**Cosm**
	***Polygonum hydropiper* L.**	**He**	**P–Herb**	**Pl**
	***Rumex conglomeratus* Murray**	**He**	**P–Herb**	**Pl**
Pottiaceae	***Barbula unguiculata* Hedw.**	**Th-Thallo-Ep**	**Br–A**	**Pl**

Primulaceae	***Primula heterochroma* Stapf**	**He**	**P–Herb**	**ES (Hyr)**
Rosaceae	*Agrimonia eupatoria* L.	He	P–Herb	Pl
	Eriobotrya japonica (Thunb.) Lindl.	Ph	Tree	Cultivated
	Geum urbanum L.	He	P–Herb	ES, IT, M
	Potentilla reptans L.	He	P–Herb	Cosm
	Rubus hyrcanus Juz.	Ch	P–Woody	ES (Hyr)
	Rubus persicus Boiss.	Ch	P–Woody	ES (Hyr)
Salicaceae	*Populus deltoides* Marshall	Ph	Tree	Cultivated
Smilacaceae	*Smilax excelsa* L.	Ph	P–Woody-Climber	ES, M
Solanaceae	***Solanum nigrum*** L.	**Th**	**A–Herb**	**Cosm**
	Solanum pseudocapsicum L.	Ch	P–Woody	Cosm
Urticaceae	***Urtica dioica* L.**	**He**	**P–Herb**	**Cosm**
Verbenaceae	*Verbena officinalis* L.	He	P–Herb	Cosm
Violaceae	*Viola odorata* L.	He	P–Herb	ES, IT, M

*__Life form:__ Ph: Phanerophyte; Ch: Chamaephyte; He: Hemichryptophyte; Ge: Geophyte; Th: Therophyte; Ep: Epiphyte.

**__Habit:__ A: Annual; P: Perennial; Herb: Herbaceous; Br: Brypophyte.

***__Chorotype:__ ES: Euro-Siberian; IT: Irano-Turanian; M: Mediteranean; Hyr: Hyrcanian; EH: Euxino-Hyrcanian; Pl: Pluriregional; Cosm: Cosmopolitan.

Table S2. List of plant species collected in all three seasons from acid soils in North Iran. The species with Al accumulation in the aerial parts (shoots) in all three seasons were indicated by an asterisk.

Species	Al accumulation in all seasons
Amaranthus blitum L.	
Amaranthus retroflexus L.	*
Barbula unguiculata Hedw.	*
Calystegia sepium (L.) R.Br.	
Campanula rapunculus L. subsp. *lambertiana* (A.DC.) Rech.f.	
Carex divulsa Stokes	
Erigeron canadensis L.	
Lamium album L.	
Mentha aquatica L.	
Mentha pulegium L.	*
Oxalis acetosella L.	
Phytolacca americana L.	
Polygonum aviculare L.	
Polygonum hydropiper L.	
Prunella vulgaris L.	
Solanum nigrum L.	*
Urtica dioica L.	

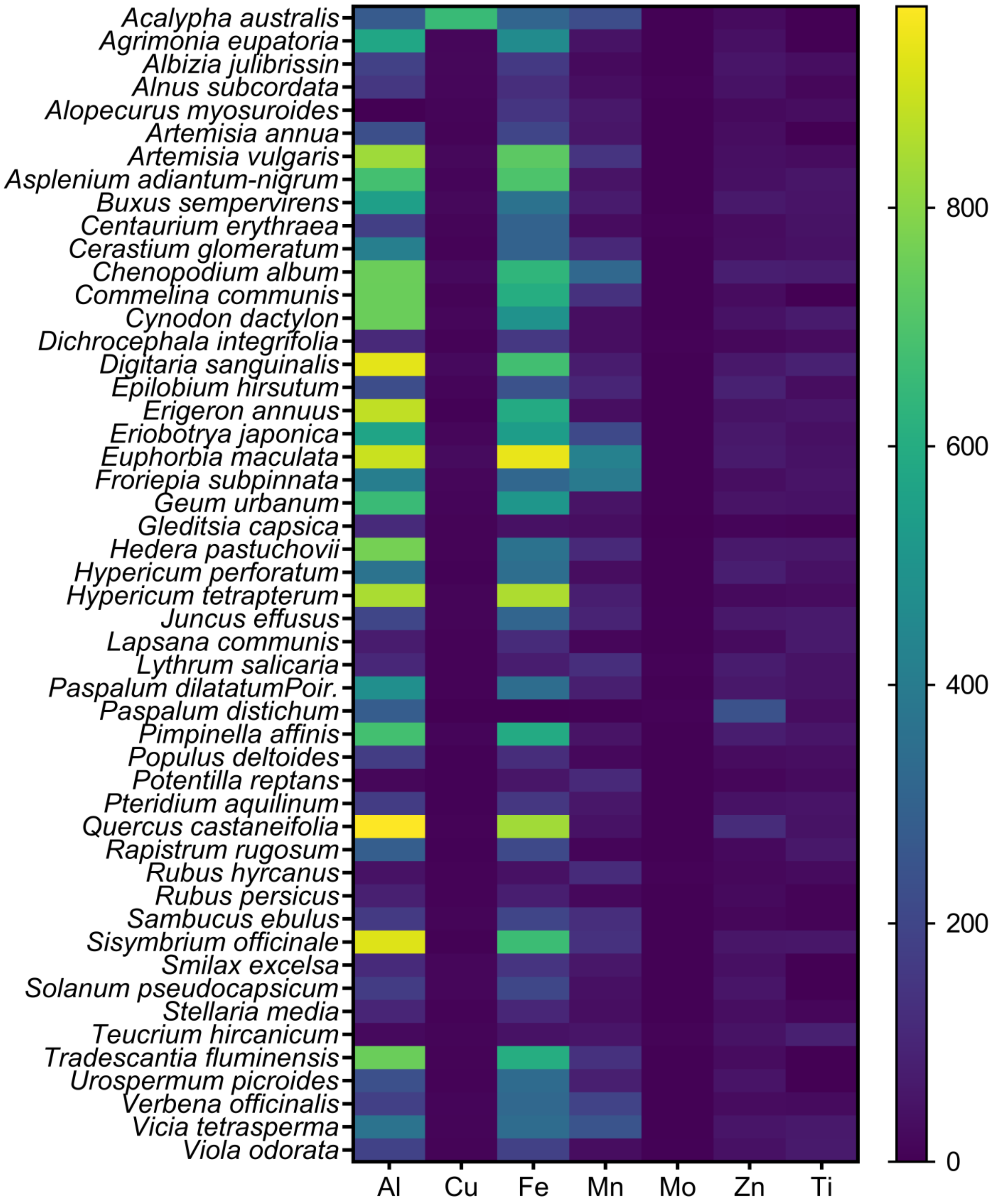

Figure S1. Heat map presenting the concentration (μg g^{-1} DW) of Al, micronutrients and Ti in the aerial parts (shoots) of excluder species.

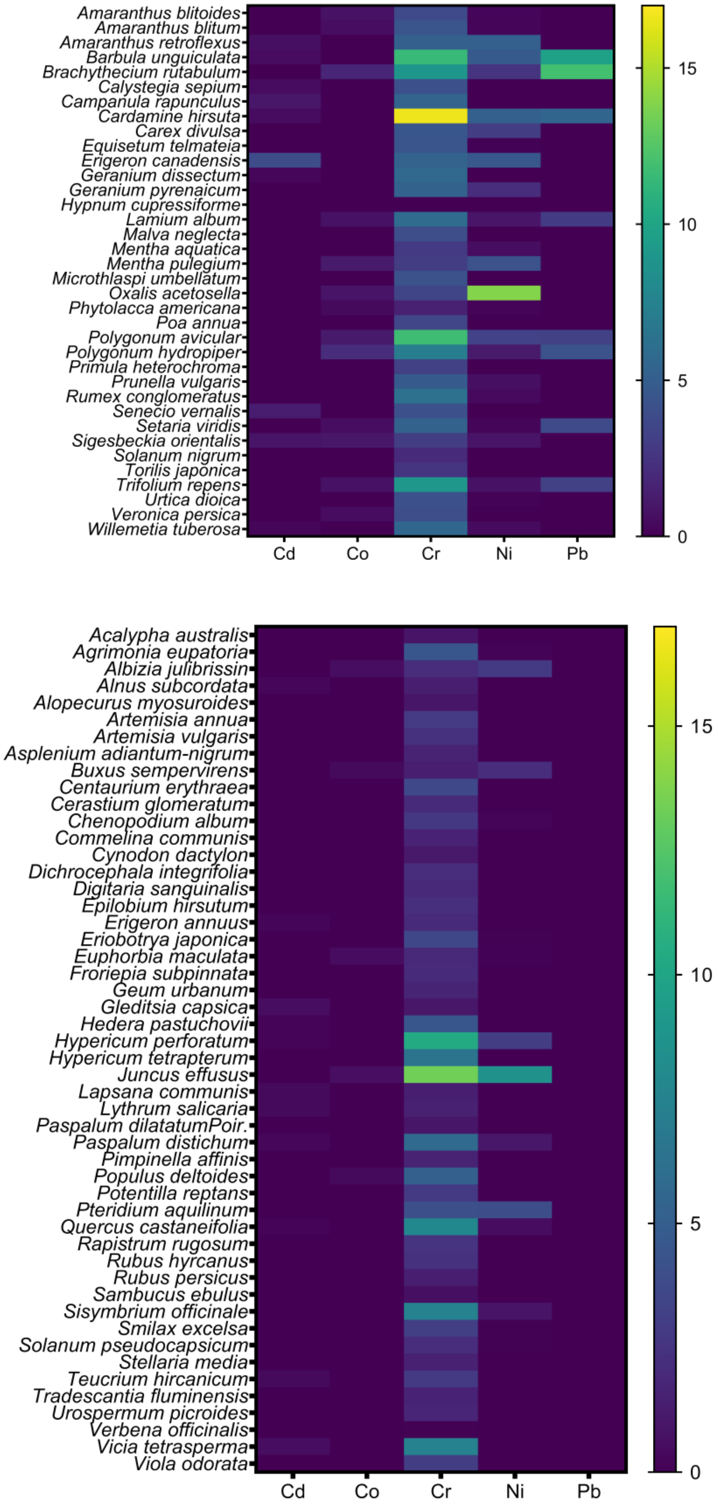

Figure S2. Heat map presenting the concentration (μg g^{-1} DW) of heavy metals in the aerial parts (shoots) of Al accumulating (above panel) and Al excluding (below panel) species.

Table S3. The difference (P-values, Mann-Whitney test) between Al accumulators and Al excluders in the concentrations of micronutrients, macronutrients, heavy metals (HM), Se and Ti in plant specimens collected from acid soils in North Iran.

Micronutrients	P values	Macronutrients+Se	P values	HM+Ti	P values
Cu	<0.001***	Ca	0.044*	Cd	0.977 ns
Fe	<0.001***	K	0.599 ns	Co	<0.001***
Mn	<0.001***	Mg	0.641 ns	Cr	<0.001***
Mo	0.015**	P	0.540 ns	Ni	<0.001***
Zn	<0.001***	S	0.141 ns	Pb	0.001***
B	0.000***	Se	0.373 ns	Ti	<0.001***

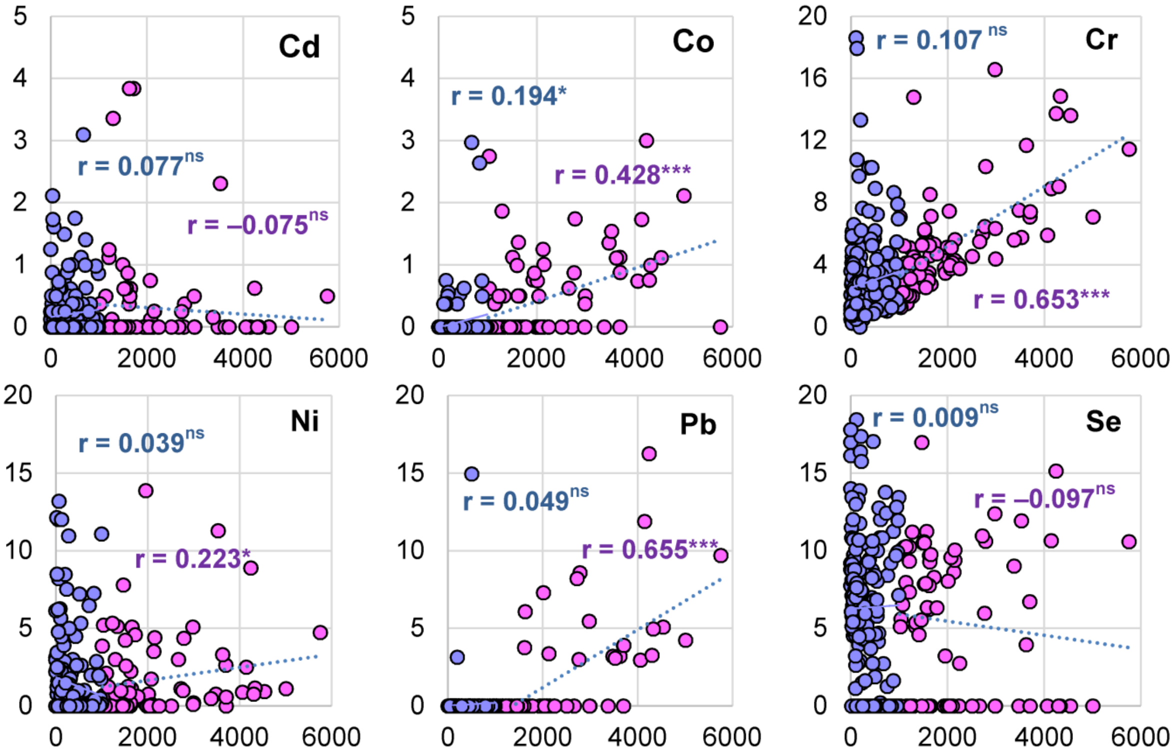

Figure S3. The correlation (Pearson's coefficient) between the concentrations of Al with heavy metals, Se and Ti in the Al-accumulating (magenta) and Al-excluding (blue) species. Significant correlations were indicated by the asterisks. *** $P<0.001$, ** $P<0.01$, * $P<0.05$, ns non significant.

Table S4. The accumulation feature for heavy metals (HM) and Al for the species designated as Al accumulators in this study. The hyperaccumulator species were listed considering the concentration threshold set for leaf concentration of each HM by Reeves et al. [44].

Species	Hyperaccumulation feature in the literature
Amaranthus blitoides S.Watson	*A. blitoides* (Pb) [45]<br>*A. tricolor* (Cd) [46]
Amaranthus blitum L.	
Amaranthus retroflexus L.	
Barbula unguiculata Hedw.	*Scopelophila cataractae* (Pottiaceae) (Cu, Fe) [47]
Brachythecium rutabulum	Not reported for HM or Al
Calystegia sepium (L.) R.Br.	*C. hederacea* (Pb) [48]
Campanula rapunculus L. subsp. *Lambertiana* (A.DC.) Rech.f.	Not reported for HM or Al
Cardamine hirsuta L.	*C. hupingshanensis* (Se) [49]; *C. violifolia* (Se) [50]
Carex divulsa Stokes	Not reported before for HM or Al
Equisetum telmateia Ehrh.	Not reported before for HM or Al
Erigeron canadensis L.	*E. canadensis* (Cu) [51] *Conyza cordata* (syn. *Erigeron cordatus*) (Cu) [52]
Geranium dissectum L.	Not reported for HM or Al
Geranium pyrenaicum Burm.f.	
Hypnum cupressiforme	*Ectropothecium zollingeri* (Hypnaceae) (Fe) [47]
Lamium album L.	Not reported for HM or Al
Malva neglecta Wallr.	*M. sinensis* (Cd) [53]
Mentha aquatica L.	Not reported for HM or Al
Mentha pulegium L.	
Microthlaspi umbellatum F.K.Mey.	Not reported for HM or Al
Oxalis acetosella L.	Not reported for HM or Al
Phytolacca americana L.	*P. acinosa* (Mn) [54], *P. americana* (Mn) [55] ***P. Americana* (Al and Mn)** [56]
Poa annua L	Not reported for HM or Al
Polygonum aviculare L.	*P. perfoliatum*, *P. hydropiper* (Mn) [57]<br>*P. lapathifolium, P. pubescens* (Mn) [58]
Polygonum hydropiper L.	
Primula heterochroma Stapf	Not reported for HM or Al
Prunella vulgaris L.	Not reported for HM or Al
Rumex conglomeratus Murray	*R. obtusifolius* (Al) [59]<br>***R. acetosa* (Al) [60]**
Senecio vernalis Waldst. & Kit.	*S. azulensis*, *S. biseriatus*, *S. ekmanii* , *S. plumbeus* , *S. riäalis*, *S. subsquarrosus* (Ni) [61]
Setaria viridis (L.) P.Beauv.	***Setaria sp* (Al) [22]**
Sigesbeckia orientalis L.	Not reported for HM or Al
Solanum nigrum L.	*S. nigrum* (Cd) [62]
Torilis japonica (Huott.) DC.	Not reported before for HM or Al
Trifolium repens L.	*T. repens* (Pb) [63]
Urtica dioica L.	Not reported for HM or Al
Veronica persica Poir.	*V. anagallis-aquatica* (Cd) [64]
Willemetia tuberosa Fisch. & C. A. Mey. Ex DC.	Not reported before for HM or Al

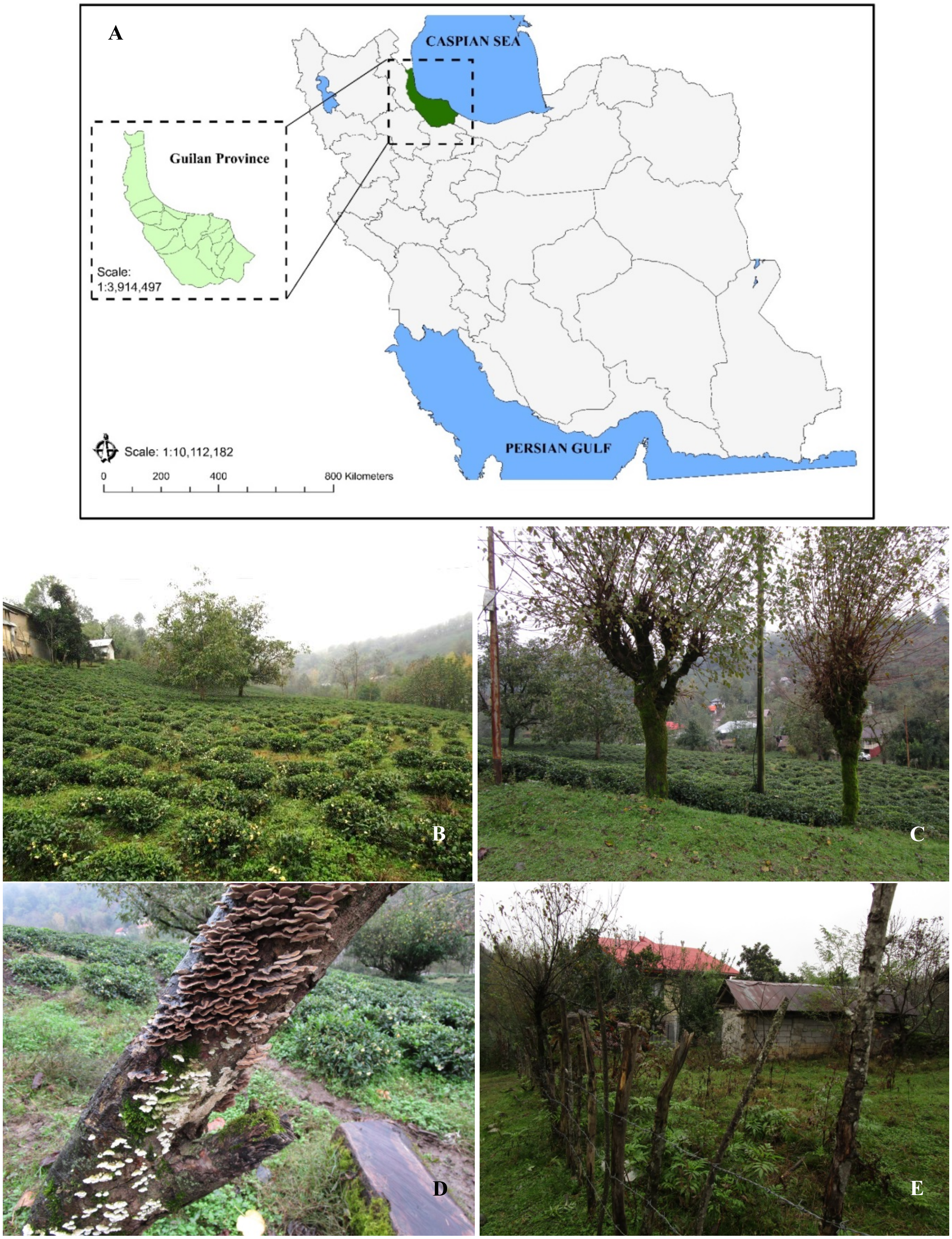

Figure S4. Iran map showing the study area in the south of the Caspian sea, North Iran (A). Pictures from the collection sites: tea gardens (B, C) and around area (D, E).

Table S5. Physical and chemical properties and the available and total concentration of the selected mineral elements in the soil samples collected from three locations in the study area*.

**Chemical and physical properties**				
Location	pH	EC (dS m^{-1})	Organic C (%)	Texture
Bakhshi	3.4	0.37	3.0	Sandy clay loam
Siahkal	4.2	0.35	2.8	Sandy clay loam
Sheikhanbar	5.4	0.22	1.9	Loam
**Available concentration of soil elements and chemical properties**				
Location	Al (mg kg^{-1})	B (mg kg^{-1})	Cu (mg kg^{-1})	Fe (mg kg^{-1})
Bakhshi	14.4	0.06	1.54	37.6
Siahkal	2.9	0.02	0.80	20.6
Sheikhanbar	1.2	0.02	0.60	5.6
	Mn (mg kg^{-1})	Mo (mg kg^{-1})	Zn (ppb)	Se (ppb)
Bakhshi	112.7	0.01	1.2	80
Siahkal	39.5	0.01	1.5	20
Sheikhanbar	21.0	0.01	0.7	20
	P (mg kg^{-1})	K (mg kg^{-1})	Cr (mg kg^{-1})	Ni (mg kg^{-1})
Bakhshi	6.6	167	0.022	0.96
Siahkal	14.3	228	0.002	0.66
Sheikhanbar	11.3	153	0.002	0.28
**Total concentration of soil elements**				
Location	Al (mg kg^{-1})	B (mg kg^{-1})	Cu (mg kg^{-1})	Fe (mg kg^{-1})
Bakhshi	4300	122	38.1	4000
Siahkal	5700	187	34.7	6300
Sheikhanbar	5200	148	20.4	4800
	Mn (%)	Mo (mg kg^{-1})	Zn (mg kg^{-1})	Se (mg kg^{-1})
Bakhshi	0.23	16.4	68.2	80.3
Siahkal	0.30	22.8	74.3	108.9
Sheikhanbar	0.12	20.7	62.9	99.9
	P (mg kg^{-1})	K (mg kg^{-1})	Cr (mg kg^{-1})	Ni (mg kg^{-1})
Bakhshi	886	620	51.8	25.8
Siahkal	899	930	72.9	38.6
Sheikhanbar	699	760	69.6	23.6
	Cd (mg kg^{-1})	Co (mg kg^{-1})	Pb (mg kg^{-1})	Ti (mg kg^{-1})
Bakhshi	n.d.	17.54	58.57	942
Siahkal	n.d.	16.83	70.37	2525
Sheikhanbar	n.d.	14.40	68.20	1484

*The physical and chemical analysis of soil was performed using the standard methods [65]. Soil pH was determined in the saturated paste. EC was determined in the water extract (1:1 soil:water). Soil texture was determined using the hydrometer method. For determination of plant available microelements (and Al), soil was extracted in a solution containing 5 mM DTPA, 10 mM $CaCl_2$, and 100 mM TEA (pH 7.3). The suspension was shaken at 120 rpm for 2 h, filtered through Whatman® and analyzed by ICP-MS (Agilent 7900, USA, Ariya Chemistry Sharif, Co, Tehran, Iran). Total organic carbon was determined by Walkley-Black chromic acid wet oxidation method. Available

phosphorus (P) and potassium (K) were determined after extraction of soil with Bray-1 and 1 M NH_4OAc (pH 7.0), respectively. The total concentration of selected mineral elements was determined after digestion in HNO_3 using ICP-OES (Spectro-Genesis EOP II; Spectro Analytical Instruments GmbH, Kleve, Germany). n.d., not detected.

Table S6. Soil chemical properties in the samples collected in three successive years and three seasons from the Siahkal Tea Research Station*.

Year and season		pH	EC (dS m^{-1})	Organic C (%)	Available P (mg kg^{-1})	Available K (mg kg^{-1})
2018	Spring	4.4	0.11	2.6	12.4	287
	Autumn	4.5	0.19	2.4	29.1	230
	Winter	4.1	0.20	1.7	70.1	309
2019	Spring	4.8	0.21	1.7	3.70	310
	Autumn	4.7	0.13	2.1	33.1	171
	Winter	4.5	0.24	2.0	15.8	234
2020	Spring	4.5	0.15	1.9	55.3	281
	Autumn	4.6	0.22	2.6	9.70	281
	Winter	4.5	0.24	2.0	15.8	234

*Data were provided by the Tea Research Center of Iran Horticultural Science Research Institute, Lahijan, Iran.

Table S7. The composition of nutrient solution (pH-4.0) and the Al concentration and the free Al^{3+} activity (calculated using GEOCHEM-PC) used for hydroponic culture of plants.

Macronutrients	mM	Micronutrients	μM
KNO_3	1.2	H_3BO_3,	5.0
$Ca(NO_3)_2.4H_2O$	0.8	$MnSO_4.H_2O$	0.4
$NH_4H_2PO_4$	0.1	$ZnSO_4.7H_2O$	0.4
$MgSO_4.7H_2O$	0.1	$CuSO_4.5H_2O$	0.1
NH_4Cl	0.3	H_2MoO_4	0.1
$MgCl_2$	0.1	Fe-EDTA	4.0
Al concentration (μM)		Free Al^{3+} activity (μM)	
0		0	
50		11.97	
400		125	

References

44. Reeves, R.D.; Baker, A.J.; Jaffré, T.; Erskine, P.D.; Echevarria, G.; van der Ent, A. A global database for plants that hyperaccumulate metal and metalloid trace elements. *New Phytol.* **2018**, *218*, 407–411.
45. Del Río-Celestino, M.; Font, R.; Moreno-Rojas, R.; De Haro-Bailón, A. Uptake of lead and zinc by wild plants growing on contaminated soils. *Ind. Crops Prod.* **2006**, *24*, 230–237.
46. Watanabe, T.; Murata, Y.; Osaki, M. *Amaranthus tricolor* has the potential for phytoremediation of cadmium-contaminated soils. *Commun. Soil Sci. Plant Anal.* **2009**, *40*, 3158–3169.
47. Printarakul, N.; Meeinkuirt, W. The bryophyte community as bioindicator of heavy metals in a waterfall outflow. *Sci. Rep.* **2022**, *12*, 1–2.
48. Sun, Z.; Chen, J.; Wang, X.; Lv, C. Heavy metal accumulation in native plants at a metallurgy waste site in rural areas of Northern China. *Ecol. Eng.* **2016**, *86*, 60–68.
49. Yuan, L.; Zhu, Y.; Lin, Z.Q.; Banuelos, G.; Li, W.; Yin, X. A novel selenocystine-accumulating plant in selenium-mine drainage area in Enshi, China. *PLoS ONE* **2013**, *8*, e65615.
50. Both, E.B.; Stonehouse, G.C.; Lima, L.W.; Fakra, S.C.; Aguirre, B.; Wangeline, A.L.; Xiang, J.; Yin, H.; Jókai, Z.; Soós, Á.; Dernovics, M. Selenium tolerance, accumulation, localization and speciation in a *Cardamine* hyperaccumulator and a non-hyperaccumulator. *Sci. Total Environ.* **2020**, *703*, 135041.
51. Boojar, M.M.; Tavakkoli, Z. New molybdenum-hyperaccumulator among plant species growing on molybdenum mine-a biochemical study on tolerance mechanism against metal toxicity. *J. Plant Nutr.* **2011**, *34*, 1532–1557.
52. van Der Ent, A.; Vinya, R.; Erskine, P.D.; Malaisse, F.; Przybyłowicz, W.J.; Barnabas, A.D.; Harris, H.H.; Mesjasz-Przybyłowicz, J. Elemental distribution and chemical speciation of copper and cobalt in three metallophytes from the copper–cobalt belt in Northern Zambia. *Metallomics* **2020**, *12*, 682–701.
53. Zhang, S.; Chen, M.; Li, T.; Xu, X.; Deng, L. A newly found cadmium accumulator—*Malva sinensis* Cavan. *J. Hazard. Mater.* **2010**, *173*, 705–759.
54. Shengguo, X.U.; Yingxu, C.H.; Qi, L.I.; Shengyou, X.; Yuanpeng, W. *Phytolacca acinosa* Roxb. (Phytolaccaceae): A new manganese hyperaccumulator plant from Southern China. *Acta Ecol. Sin.* **2003**, *23*, 935–937.

55. Min, Y.; Meizhen, T.; Aoyama, I. Accumulation and uptake of manganese in a hyperaccumulator *Phytolacca americana*. *Miner. Eng*. **2007**, *20*, 188–190.
56. Liu, P.; Tang, X.; Gong, C.; Xu, G. Manganese tolerance and accumulation in six Mn hyperaccumulators or accumulators. *Plant Soil* **2010**, *335*, 385–395.
57. Liu, C.; Liu, W.S.; van der Ent, A.; Morel, J.L.; Zheng, H.X.; Wang, G.B.; Tang, Y.T.; Qiu, R.L. Simultaneous hyperaccumulation of rare earth elements, manganese and aluminum in *Phytolacca americana* in response to soil properties. *Chemosphere* **2021**, *282*, 131096.
58. Deng, H.; Li, M.S.; Chen, Y.X. Accumulating characteristics of manganese by *Polygonum pubescens* Blume. *Acta Ecol. Sin*. **2009**, *29*, 5450–5454.
59. Vondráčková, S.; Száková, J.; Drábek, O.; Tejnecký, V.; Hejcman, M.; Müllerová, V.; Tlustoš, P. Aluminium uptake and translocation in Al hyperaccumulator *Rumex obtusifolius* is affected by low-molecular-weight organic acids content and soil pH. *PLoS ONE* **2015**, *10*, e0123351.
60. Tolrà, R.P.; Poschenrieder, C.; Luppi, B.; Barceló, J. Aluminium-induced changes in the profiles of both organic acids and phenolic substances underlie Al tolerance in *Rumex acetosa* L. *Environ. Exp. Bot.* **2005**, *54*, 231–238.
61. Reeves, R.D.; Baker, A.J.; Borhidi, A.; Berazain, R. Nickel hyperaccumulation in the serpentine flora of Cuba. *Ann. Bot*. **1999**, *83*, 29–38.
62. Wei, S.H.; Zhou, Q.X.; Wang, X. Cadmium-hyperaccumulator *Solanum nigrum* L. and its accumulating characteristics. *Huan Jing Ke Xue* **2005**, *26*, 167–171. (In Chinese)
63. Bech, J.; Roca, N.; Tume, P.; Ramos-Miras, J.; Gil, C.; Boluda, R. Screening for new accumulator plants in potential hazards elements polluted soil surrounding Peruvian mine tailings. *Catena* **2016**, *136*, 66–73.
64. Hadi, F.; Ahmad, A.; Ali, N. Cadmium (Cd) removal from saline water by *Veronica anagallis* and *Epilobium laxum* plants in hydroponic system. *Agric. Sci.* **2014**, *5*, 935.
65. Sparks, D.L.; Page, A.L.; Helmke, P.A.; Loeppert, R.H., (Eds.). *Methods of Soil Analysis; Part 3: Chemical Methods*; John Wiley & Sons: Hoboken, NJ, USA, 2020; ISBN: 978-0-891-18825-4.
